# Efficacy of Azoxystrobin on Mycotoxins and Related Fungi in Italian Paddy Rice

**DOI:** 10.3390/toxins11060310

**Published:** 2019-05-30

**Authors:** Paola Giorni, Umberto Rolla, Marco Romani, Annalisa Mulazzi, Terenzio Bertuzzi

**Affiliations:** 1 Department of Sustainable Crop Production—DIPROVES, Faculty of Agriculture, Food and Environmental Science, Università Cattolica del Sacro Cuore, via Emilia Parmense 84, 29122 Piacenza, Italy; 2 Ente Nazionale Risi, Rice Research Centre, Strada per Ceretto 4, Castello d’Agogna, 27030 Pavia, Italy; u.rolla@enterisi.it (U.R.); m.romani@enterisi.it (M.R.); 3 Department of Animal, Food and Nutrition Science—DIANA, Università Cattolica del Sacro Cuore, Via Emilia Parmense 84, 29122 Piacenza, Italy; annalisa.mulazzi@unicatt.it (A.M.); terenzio.bertuzzi@unicatt.it (T.B.)

**Keywords:** rice, mycotoxins, sterigmatocystin, STC, deoxynivalenol, DON, growing season, azoxystrobin, fungicide

## Abstract

The efficacy of azoxystrobin was evaluated in the presence of mycotoxigenic fungi and relative mycotoxins in Italian paddy rice during the growing season in the field. Three experimental fields were considered and the applied experimental design was a strip plot with three replicates; rice samples were collected at four different growing stages. The efficacy of the fungicide treatment on rice fungal population was demonstrated with around 20% less total fungal incidence in sprayed samples compared to untreated ones; the same decrease was noted also in *Fusarium* spp. species but not in *Aspergillus versicolor*. Of the mycotoxins considered, ochratoxin A (OTA) and aflatoxins (AFBs) were never detected, deoxynivalenol (DON) was found in 46% of samples at levels always lower than 100 µg/kg, while sterigmatocystin (STC) occurred in all the paddy rice samples collected after flowering, with a maximum value of 15.5 µg/kg. Treatment with azoxystrobin was not effective in reducing DON contamination, but it had an important and significant effect on STC content, showing a decrease of 67% in the sprayed samples.

## 1. Introduction

Rice (*Oryza sativa* L.) is the staple food for almost half of the world’s population [1]; it is cultivated mainly in Asian regions and China is the largest producer. The main rice producer in Europe is Italy, accounting for around 50% of total European production. Rice cultivation is principally in Northern Italy (Piedmont and Lombardy) and is destined for several food uses, baby foods included.

Different diseases can affect rice and, in particular, fungi can be particularly dangerous for plant and grain health [2] during both the growing season and post-harvest [3]. Panicle blast caused by *Pyricularia grisea* [4] and brown spot caused by *Bipolaris oryzae* [5] are the most dangerous diseases for Italian rice crops, occurring frequently and causing production and economic losses [4]. Nowadays, particular attention must also be paid to fungal species which can produce, in favorable environmental and substrate conditions, various mycotoxins that can impact human health. The presence of mycotoxigenic fungi on paddy rice has been indicated in several reports; in particular, *Fusarium* spp., responsible for trichothecenes (expecially deoxynivalenol (DON)) and fumonisins (FBs) [6,7], *Aspergillus flavus*, able to produce aflatoxins (AFs) [8], and *Penicillium* spp. for ochratoxin A (OTA) and citrinin (CIT) [9]. Recently, sterigmatocystin (STC) produced by *Aspergillus versicolor* was also found in rice and resulted the most common mycotoxin in Italian rice [10]. The European Commission fixed strict limits for mycotoxins in cereals; in particular, for rice destined for human consumption limits are present for AFs (2.0 µg/kg), OTA (3.0 µg/kg), and DON (750 µg/kg), making mycotoxin containment very important for product exchanges (EU Regulation 165/2010; EC Regulation 1881/2006; EU Regulation 1006/2015).

Among possible strategies to control mycotoxigenic fungi development in the field and, consequently, mycotoxin production, fungicides can be used. Negative effects have been reported in some cases, such as the reduction of beneficial microorganisms for plant growth due to acidification of the soil [11] or the possible selection of fungicide-resistant fungal strains [12]. Moreover, each toxigenic fungal species responds differently to fungicides because several factors can contribute to their reaction; in particular, weather, active ingredients, plant development stage, and cultivar resistance can play a role [13,14] and can act as stressors in the production of mycotoxins [15]. For example, it has been found that triazole applications can reduce both *F. graminearum* and DON occurrence [16,17], especially if the treatments are carried out before fungal infection [11]. However, in some cases the use of fungicide can increase mycotoxin content; Dors et al. [15] reported that tebuconazole was able to act as an elicitor of stress for mycotoxigenic fungi and, consequently, enhanced the presence of several mycotoxins.

There are few fungicides allowed by Italian Regulations for rice; of these, azoxystrobin is an active ingredient belonging to the strobilurin chemical group and it is one of the most used on Italian rice because of its demonstrated efficacy on several crops and its major role in reducing *Pyricularia grisea* [18] and *Bipolaris oryzae* infections in rice [19] and Fusarium Head Blight (FHB) in wheat [20]. However, its possible effect on mycotoxins is still uncertain since in some studies the use of azoxystrobin in wheat could result in an increase in DON content up to 42% [21,22]; for this reason, this possible effect needs to be evaluated also in rice in order to assist farmers in their selection of fungicides.

The aim of this study was to define the efficacy of azoxystrobin on mycotoxigenic fungal species present in paddy rice during the growing season from flowering to over ripening (1 June–30 September) and determine its possible effect on the production of their relative mycotoxins. 

## 2. Results and Discussion

### 2.1. Efficacy of Azoxystrobin on Mycotoxigenic Fungi

The highest fungal incidence was found at the full ripening stage with more than 70% of the rice kernels infected (Table 1). Fungi seem to increase their incidence throughout the growing season up to ripening, then they significantly decrease if left in field for an additional 14 days obtaining around a 10% reduction for total fungi incidence (Table 1). The same level of reduction was not observed for mycotoxigenic species that reach their maximum incidence at harvest time (full ripening) and maintain their presence even in the case of over ripening. Both for *Fusarium* spp. and *A. versicolor*, the only mycotoxigenic species resulting with a significant presence in field, no differences were found between the full ripening and over-ripening stages (Table 1).

The same was found in a previous study on paddy rice [10] with the only exception of *Fusarium* spp. that seemed to decrease in over ripening in accordance with the total fungi trend; probably, different meteorological conditions registered after full ripening, in particular the almost total absence of rain observed in the area in year 2018, could have influenced *Fusarium* spp. vitality.

As expected, different rice varieties showed different levels of fungal contamination; in particular, Terra CL showed the highest fungal content while CL26 the lowest (Table 1). *Fusarium* spp. exhibited the same trend while *A. versicolor* presence resulted always very low and with no significant differences between rice varieties. However, interestingly, *A. versicolor*, differently from other fungal species, showed a higher incidence in CL26, which was the rice variety least contaminated by other fungal species, and a lower incidence in Terra CL and CL15 varieties which were the most contaminated by other fungal species (Table 1). This was probably due to varying fungal abilities to compete in extreme environmental conditions; the year 2018, in fact, was notable in Italian rice cultivation areas for an almost total absence of rain (total rainfall was only 157.4 mm in the period 1 June–30 September) and extreme temperatures (up to 36 °C). This was undoubtedly favorable for xerophilic species, such as *A. versicolor* [23], in particular on rice varieties where fungal incidence and, as a consequence, fungal competition were lower.

The efficacy of the fungicide treatment on rice fungal population was demonstrated with around 20% less total fungal incidence in sprayed samples than the untreated ones (Table 1). The same decrease was noted also in the *Fusarium* spp. species but not in *A. versicolor* which was unchanged (Table 1). The effect of strobilurins against fungi is well documented, they appear able to enhance rice plant defenses against pathogen attacks [19,24], shown also in wheat against mycotoxigenic *Fusarium* species like *F. graminearum* [20]. The incidence of *A. versicolor* was too low to obtain a significant reduction in treatment with azoxystrobin, although a reduction of 5% was observed.

Differences in fungal contamination were found between the three different experimental fields; in particular, experimental field C was the most contaminated with also the highest *Fusarium* spp. incidence. No significant differences were found between experimental fields in the presence of *A. versicolor*.

### 2.2. Efficacy of Azoxystrobin on Mycotoxin Production

Among the considered mycotoxins considered, OTA and AFs were never detected; DON was found in 46% of the samples at levels always lower than 100 µg/kg, while STC occurred in almost all the paddy rice samples, showing a maximum value of 15.5 µg/kg. These data partially accord with a previous study, carried out on rice samples collected in the same area, that found DON and AFs only sporadically and in low amounts, while STC was always detected, appearing as crucial in rice contamination [10].

Regarding mycotoxin accumulation, it is important to note that both DON and STC follow the same trend of their producing fungi. In particular, DON was highest at full ripening and remained constant up to over ripening as happened for *Fusarium* spp. fungi, while STC was highest at full ripening and significantly decreased in over-ripening, as happened to the presence of *A. versicolor* (Table 1). A similar result for STC was found in a previous research, even if this decrease was not so intensive [10]. This could be due to environmental and substrate conditions that probably reduce fungal ability to produce STC while they have no effect on DON production. Significant differences in mycotoxin contamination were found between rice varieties with Sole CL resulting one of the most contaminated by DON and the one with the highest STC content (Table 1).

The highest DON contamination was found in the rice variety CL15 while the lowest was in the rice variety Centauro (Table 1); none of the rice varieties considered in the study showed a DON contamination above the limits fixed by the European Commission of 1250 µg/Kg for paddy rice (EU Regulation 1881/2006). These results seem to confirm the findings of a previous study where DON was found only in low amounts [10] suggesting that this mycotoxin could be considered a minor risk for Italian paddy rice.

**Figure 1:**
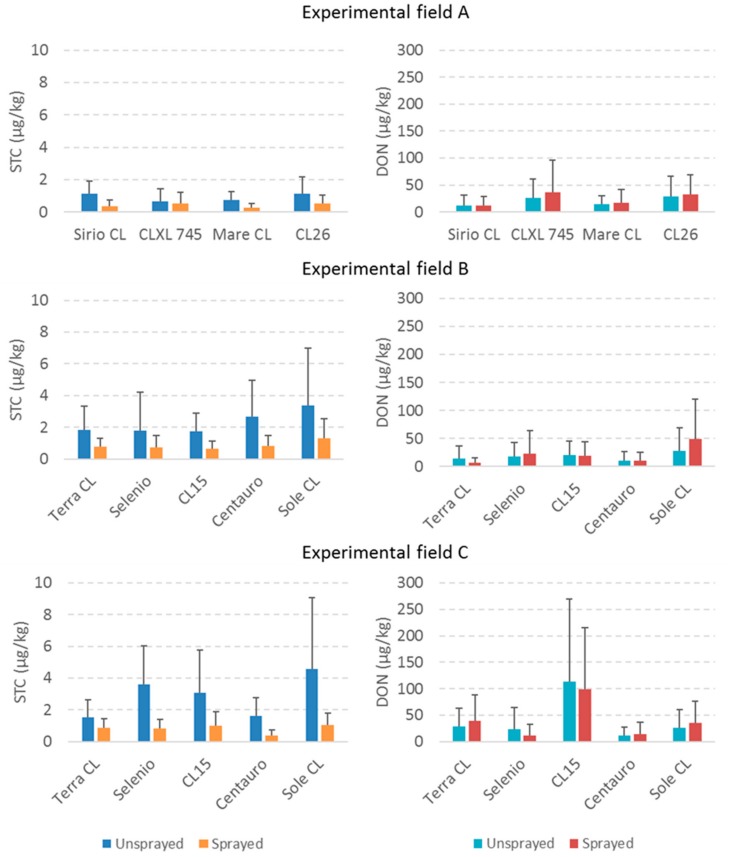
Mean sterigmatocystin (STC) and deoxynivalenol (DON) contamination in the different experimental fields considered in the trial in case of unsprayed and sprayed paddy rice varieties.

The treatment with azoxystrobin was inefficient in reducing DON contamination; contrarily, DON increased in sprayed samples even if the results were not statistically different (Figure 1). The fungicide had an important and significant effect on STC content, showing a decrease of 67% in the sprayed samples (Table 1). These data partially agree with previous findings on wheat, where the use of strobilurin obtained a good reduction of FHB, but with an uncertain impact on DON reduction [20,25]. The results obtained in STC reduction are very promising because this mycotoxin seems to be the most dangerous for Italian rice production and treatment with azoxystrobin, one of the active ingredients allowed by the Italian government on rice, could be useful for reducing STC contamination in field during the growing season.

Significant differences in mycotoxin contamination were observed between experimental fields (*p* ≤ 0.01); in particular, experimental field C was the most contaminated having the highest incidence of total fungi. Moreover, the same experimental field showed the highest STC content (1.9 µg/Kg vs 0.7–1.6 µg/Kg) and the highest DON content (40 µg/Kg vs 20–22 µg/Kg) (Table 1). Differences in mycotoxins contamination between experimental fields were expected since many variables can contribute to their presence such as susceptibility of rice variety, preceding crop, tillage, and pest presence [26,27]. However, even if we tried to keep the differences in agronomic management minimal between experimental fields, environmental factors, such as relative humidity and temperature, can always play a relevant and unpredictable role in both fungal contamination and mycotoxin occurrence.

Considering treatment with azoxystrobin on single rice varieties, important reductions were observed. In particular, Sole showed the highest STC reduction being, respectively, of 62% and 77% in experimental field B and C (Figure 1). The lowest reduction (22%) in STC obtained in sprayed samples was found in the rice variety CLXL745 (Figure 1). Differences in azoxystrobin efficacy between rice varieties were probably due to their different susceptibility to fungicide, as already found in other studies with other active ingredients [28] and on other crops [29,30].

## 3. Conclusions

At first, this study confirmed that *Fusarium* spp. and *A. versicolor* were the most frequently mycotoxigenic species found on Italian paddy rice during the growing season. As a consequence, DON and STC can occur in paddy rice samples at different plant growing stages. Treatment with azoxystrobin as an active ingredient seems to be efficient in reducing total fungi and *Fusarium* spp. incidence and STC contamination, while they have no effect on *A. versicolor* presence and DON level. Rice varieties played an important role for their different and proven susceptibility to fungal diseases and fungicide efficacy.

STC, an emerging mycotoxin that is not routinely checked, has been confirmed as a relevant mycotoxin in paddy rice, confirming a previous survey that collected rice samples of different origin [31]; the contamination level of this mycotoxin could be considered for future EU legislative regulation.

The results obtained showed that the use of azoxystrobin could help farmers to develop a potential method for STC containment in conditions particularly conducive for fungal development and mycotoxin production, being necessarily cautious in their use due to uncertain reduction of the presence of DON.

## 4. Materials and Methods

### 4.1. Field Samples

Sampling of rice was conducted at four different growing stages (flowering (BBCH 69), early dough (BBCH 83), ripening (BBCH 89), and over-ripening (BBCH 92, 15 days post-ripening) in 2018 in three experimental fields located close to Mortara (PV) in Lombardy, the main Italian rice production region. Nine rice varieties, both long B and round grain were considered (Table 1). In experimental field A were cultivated four long B grain rice varieties (CLXL 745, CL26, Sirio CL, Mare CL) while five common round grain rice varieties (Sole CL, Selenio, Centauro, Terra CL, CL15) were cultivated in experimental fields B and C. Soil texture and sowing period varied, while the meteorological conditions can be considered similar because of the proximity of the fields (all within 10 km).

### 4.2. Fungicide Treatment

The applied experimental design was a strip plot with three replicates; each strip (6 m × 100 m), containing rice plants of 1 variety with a sowing density of 150 kg/ha of seeds, was subdivided into three plots considered as replicates. Working at field level, it was not possible to apply a randomized plot design.

In each experimental field, two assays were considered for each rice variety: “unsprayed strip” used as control, where no fungicide was sprayed, and “sprayed strips” where treatment with fungicide was carried out. The distance between “unsprayed” and “sprayed” strips was 20 meters, which was considered sufficient to prevent problems linked to spray drift and possible contamination between the two considered assays.

Commercial formulations with azoxystrobin in the same concentration (250 g/L) were distributed in open field, on the whole width of plots chosen as “sprayed”, using a backpack sprayer (mod. SP 126, Oleo-Mac Bagnolo in Piano, Reggio Emilia, Italy) calibrated to spread 250 L/ha of solution. In all the sprayed experimental assays, only 1 fungicide treatment was scheduled when rice plants were at the stage of panicle emergence (BBCH 51-55). We decided to do the fungicide treatment at this plant growing stage because this time is normally chosen also for the scheduled paddy rice treatment against the main pathogens such as *Pyricularia grisea* and *Bipolaris oryzae*.

For each rice variety and experimental field, rice plants were collected from each plot with an X-shape design, then the plants were shelled and the grains obtained considered as representative. For each plot, representing a replicate, 500 g of grains were randomly chosen as sample. Samples were used for mycological analyses and then dried, milled using a cyclone hammer mill (1 mm sieve, Pulverisette, Fritsch GmbH, Idar-Oberstein, Germany), homogenized and kept at 4 °C until chemical analysis.

### 4.3. Monitoring of Mycotoxigenic Fungi

Fifty kernels were randomly selected from each sample, surface disinfected in 1% sodium hypochlorite for 2 min and in 90% ethyl alcohol for 2 min and then transferred onto Petri dishes containing potato dextrose agar (PDA, Biolife, Milano, Italy). The Petri dishes were incubated at 25 °C (12 h light photoperiod) and after 5–7 days the incidence of kernels infected by fungi was quantified. *Fusarium* spp. and *Penicillium* spp. isolates were identified at Genus level thanks to observations with binocular microscope (40×); only *Aspergillus* spp. isolates were identified at species level observing their morphological characteristics with magnification between 100× and 400× according to Raper and Fennell [32].

### 4.4. Monitoring of Mycotoxins

The analyses were carried out using the following methods: AFs were determined by HPLC-FLD (liquid chromatography with fluorimeter detector) as reported by Bertuzzi et al. [33]; OTA by HPLC-FLD [34], DON by GC-MS (gas chromatography coupled to mass spectrometer) [35], STC by LC-MS/MS (liquid chromatography coupled to mass spectrometer) [36]. The analyses were recently described in the work of Bertuzzi et al. [10].

### 4.5. Data Analysis

The data were transformed before statistical analysis; in particular, fungal incidence was arcsine transformed and mycotoxin content was ln transformed [37]. Analysis of variance (ANOVA) was calculated using the generalized linear model (GLM) procedure of the statistical package IBM SPSS Statistics 21 (IBM Corp., Armonk, NY, USA) while significant differences were highlighted using the Tukey test (*p* ≤ 0.05) for mean separation.

## Figures and Tables

**Table 1:** Analysis of variance (ANOVA) of fungal incidence and contamination of sterigmatocystin (STC) and deoxynivalenol (DON) at different sampling times in different rice varieties sprayed or unsprayed with fungicides formulated with azoxystrobyn (250 g/L) in three different experimental fields. Data refer to mean data; all experiments were conducted with three replicates.

	Total Fungi Incidence (%)		Incidence of *Fusarium* spp. (%)		Incidence of *A. versicolor* (%)		STC (µg/kg)		DON (µg/kg)	
**Sampling time (A)**	**		**		**		**		**	
Flowering (BBCH 69)	4.6	d	1.1	c	0.0	b	0.0	d	0.0	c
Early dough (BBCH 83)	33.3	c	7.8	b	0.1	b	1.3	c	14.4	b
Full Ripening (BBCH 89)	73.2	a	14.1	a	1.2	a	2.8	a	62.1	a
Over ripening (BBCH 92)	61.6	b	14.9	a	1.1	a	1.5	b	35.2	a
**Rice variety (B)**	**		**		n.s.		**		**	
Sirio CL	48.9	ab	9.2	abc	1.2		0.7	cd	11.8	ab
CLXL 745	37.8	cd	9.0	bcd	1.3		0.6	d	31.7	ab
Mare CL	31.2	de	6.6	cd	0.8		0.5	d	15.2	ab
CL26	26.7	e	5.5	d	1.6		0.8	c	30.8	ab
Terra CL	53.6	a	12.0	a	0.2		1.2	b	22.5	ab
Selenio	41.3	bcd	11.3	ab	0.3		1.7	b	19.1	ab
CL15	45.7	abc	10.5	abc	0.2		1.6	b	63.0	a
Centauro	42.4	bc	7.5	bcd	0.5		1.4	b	11.3	b
Sole CL	47.0	abc	9.9	abc	0.6		2.6	a	34.5	ab
**Fungicide (C)**	**		**		n.s.		**		n.s.	
Unsprayed	47.6	a	10.5	a	0.61		2.1	a	26.8	
Sprayed	38.7	b	8.4	b	0.58		0.7	b	29.0	
**Experimental field (D)**	**		**		n.s.		**		*	
A	36.1	b	7.6	c	1.2		0.7	c	22.4	b
B	38.0	b	9.6	b	0.4		1.6	b	20.0	b
C	54.0	a	10.9	a	0.3		1.9	a	40.2	a

Different letters mean significant differences according to Tukey Test; n.s.: not significative; *: *p* ≤ 0.05; **: *p* ≤ 0.01.
